# Computational drug repositioning for peripheral arterial disease: prediction of anti-inflammatory and pro-angiogenic therapeutics

**DOI:** 10.3389/fphar.2015.00179

**Published:** 2015-08-25

**Authors:** Liang-Hui Chu, Brian H. Annex, Aleksander S. Popel

**Affiliations:** ^1^Department of Biomedical Engineering, School of Medicine, Johns Hopkins UniversityBaltimore, MD, USA; ^2^Division of Cardiovascular Medicine, Department of Medicine and Robert M. Berne Cardiovascular Research Center, University of Virginia School of MedicineCharlottesville, VA, USA

**Keywords:** peripheral arterial disease, computational drug repositioning, inflammation, angiogenesis, drug-target network, bioinformatics, cardiovascular disease

## Abstract

Peripheral arterial disease (PAD) results from atherosclerosis that leads to blocked arteries and reduced blood flow, most commonly in the arteries of the legs. PAD clinical trials to induce angiogenesis to improve blood flow conducted in the last decade have not succeeded. We have recently constructed PADPIN, protein-protein interaction network (PIN) of PAD, and here we combine it with the drug-target relations to identify potential drug targets for PAD. Specifically, the proteins in the PADPIN were classified as belonging to the angiome, immunome, and arteriome, characterizing the processes of angiogenesis, immune response/inflammation, and arteriogenesis, respectively. Using the network-based approach we predict the candidate drugs for repositioning that have potential applications to PAD. By compiling the drug information in two drug databases DrugBank and PharmGKB, we predict FDA-approved drugs whose targets are the proteins annotated as anti-angiogenic and pro-inflammatory, respectively. Examples of pro-angiogenic drugs are carvedilol and urokinase. Examples of anti-inflammatory drugs are ACE inhibitors and maraviroc. This is the first computational drug repositioning study for PAD.

## Introduction

Recent pharmaceutical research and development (R&D) reports show that the probability of success for a new pharmaceutical compound to get to the market has declined in the last 10 years (Pammolli et al., 2011). The average time of drug development has increased from 9.7 years during the 1990s to 13.9 years from 2000 onwards. The average probability of success of total numbers of R&D projects in the cardiovascular system is only 4.86%. Drug repositioning, new use of old drugs, can shorten the development time and provide solutions for the high cost and declined number of new successful drugs of the pharmaceutical companies (Dudley et al., 2011). Computational repositioning strategies can predict new therapeutic indications for FDA-approved drugs, which then have to undergo clinical trials for the new indication (Belch et al., 2003; Ostchega et al., 2007; Shameer et al., 2015). In this study, we primarily use the network-based approach in computational drug repositioning.

Peripheral arterial disease (PAD) results from atherosclerosis, the plaque built-up inside the arteries, which blocks the blood flow in the peripheral arteries and most commonly in the arteries that perfuse the legs (Belch et al., 2003; Annex, 2013). Age, diabetes, and cigarette smoking are the major risk factors for the development of PAD (Belch et al., 2003; Ostchega et al., 2007; Annex, 2013). There are 8–12 million people with PAD in the United States (Writing Group et al., 2010). The clinical manifestations of PAD range from patients who do not report leg pain but have a lower functional capacity (approximately 50% of all PAD subjects) to patients who have intermittent claudication manifested as leg pain with walking/exercise that is relieved with rest (approximately 33–40% of all PAD subjects) (Hirsch et al., 2006; Norgren et al., 2007). With the goal to increase blood flow around blockages, clinical trials using drugs and gene delivery for therapeutic angiogenesis such as VEGF (vascular endothelial growth factor) gene delivery have been performed for the last two decades but have not been successful.

Hoier et al. showed that there was no difference in basal skeletal muscle VEGF mRNA content before and after passive or active exercise between PAD patients and control (Hoier et al., 2013). However, the basal level of anti-angiogenic protein thrombospondin-1 (TSP1) was remarkably higher in the PAD patients than control groups. They conclude that the anti-angiogenic factors dominate the pro-angiogenic factors in PAD patients. The up-regulation of TSP1 has been shown in various gene expression microarray studies of mouse (Chu et al., 2015) and human samples of PAD (Fu et al., 2008; Masud et al., 2012). Currently there are no FDA-approved drugs targeting TSP1. Therefore, the computational drug repositioning approach to predict the drugs targeting other endogenous anti-angiogenic proteins should be helpful for designing clinical trials for therapeutic angiogenesis in PAD.

Inflammation plays an important role in initiation and progression of PAD, and many circulating biomarkers such as matrix metalloproteinases (MMPs) and interleukin are considered as the clinical manifestation of PAD (Signorelli et al., 2014). Atherosclerosis is the dominant cause of many cardiovascular diseases, including myocardial infarction, heart failure, coronary artery disease (CAD), and stroke (Frostegård, 2013). Atherosclerosis is a chronic inflammatory condition. Potential anti-inflammatory treatments in atherosclerosis are reviewed in Frostegård (2013). The interplay between inflammation and endothelial progenitor cells is critical in cardiovascular diseases (Grisar et al., 2011). Combination of anti-inflammatory and pro-angiogenic treatments for PAD was suggested and validated *in vivo* by Zachman et al. (2014). However, a systematic bioinformatics approach to identify the potential drug repositioning for inhibition of anti-angiogenic and pro-inflammatory proteins for PAD is still lacking.

We previously constructed the PADPIN, protein-protein interaction network (PIN) in PAD that includes angiome, immunome, and arteriome, characterizing the processes of angiogenesis, immune response/inflammation and arteriogenesis, respectively (Chu et al., 2015). We have analyzed several available microarray gene expression datasets from ischemic and non-ischemic muscles in two mouse models of PAD (in C57BL/6 and BALB/c mouse species) from Hazarika et al. (2013) to identify important genes/proteins in PAD, such as THBS1 (thrombospondin-1), TLR4 (toll-like receptor 4), EphA4 (EPH receptor A4), and TSPAN7 (tetraspanin 7). However, none of the four genes (THBS1, TLR4, EphA4, and TSPAN7) have FDA-approved drugs to target them. Considering the time (>10 years) and cost (>$1 billion) for developing a new drug agent, drug repositioning in PAD offers promise of providing effective therapeutics in shorter time and at lower cost compared to conventional *de-novo* drug discovery and development. In addition, drug repurposing is an approach of taking agents in development that have achieved adequate safety for one indication but are tested for efficacy in another when safety is already evident.

## Materials and methods

### Resources for drugs and drug-target interactions

We rely on two major resources for drug information and drug-target, DrugBank 3.0 http://www.drugbank.ca/ (Knox et al., 2011) and Pharmacogenomics Knowledge Base (PharmGKB) http://www.pharmgkb.org/ (Whirl-Carrillo et al., 2012). DrugBank contains extensive omics data, such as pharmacogenomic, pharmacoproteomic, and pharmacometabolomic data. We use DTome (Drug-Target interactome tool) (Sun et al., 2012) to compile all the drugs included in DrugBank 3.0 (Knox et al., 2011), including the approved, experimental, nutraceutical, illicit, and withdrawn drugs. We compile three binary relations in DrugBank from DTome: drug-drug, drug-gene, and drug-target interactions. This compilation provides the rich resources for the potential repositioning or repurposing. By considering the drug safety and development time, we focus on FDA-approved drugs in this study. We compiled the three binary relations from PharmGKB: gene-disease, gene-drug, and gene-gene interactions. The drug-target interactions were compiled from both DrugBank (Knox et al., 2011) and PharmGKB (Whirl-Carrillo et al., 2012).

### Proteins in PADPIN and therapeutic angiogenesis in PAD

Details of the construction of PADPIN, protein-protein interaction (PIN) of PAD in angiogenesis, immune response and arteriogenesis, are described inChu et al. (2015). The methodology is similar to that used for constructing the global PIN of angiogenesis (angiome) that comprises 1233 proteins and 5726 interactions (Chu et al., 2012). The PIN of immune response (immunome) comprises 3490 proteins and 21,164 interactions. The PIN of arteriogenesis (arteriome) comprises 289 proteins and 803 interactions. The degree of node represents the number of links to a node in the network. The network parameter was calculated by NetworkAnalyzer (Assenov et al., 2008) in Cytoscape (Smoot et al., 2011). We start with the genes listed in the three PINs, to find the interactive drugs from the DrugBank and PharmGKB. Note that in bioinformatics publications, and specifically in protein-protein networks publications, the terms “gene” and “protein” are sometimes used interchangeably; while we mostly use “protein” term in this context, we sometime use “gene” to be consistent with previous publications.

### List of anti-angiogenic and pro-inflammatory genes

The activation of a specific biological process can be implemented using two strategies. One is direct activation of the genes involved in positive regulation of that biological process; the other is inhibition of the genes involved in negative regulation of that biological process. Specifically for PAD, to stimulate vascular growth and remodeling and increase the blood flow, we propose inhibition of genes annotated as negative regulation of angiogenesis as a therapeutic approach to stimulating angiogenesis. The rationale for this approach is that numerous clinical trials aimed at stimulating angiogenesis by growth factors such as VEGF-A and FGF-2 have not been successful. We identified 39 anti-angiogenic genes, chosen by Gene Ontology (GO: 0016525) and literature (Chu et al., 2014). The endothelial dysfunction in patients with PAD is characterized by impaired nitric oxide signaling, excessive inflammation and diminished response to angiogenic factors (Annex, 2013). To inhibit the inflammation, we propose inhibition of pro-inflammatory responses as a therapeutic approach for anti-inflammatory treatment of PAD. There are 89 genes classified in positive regulation of inflammatory response (GO:0050729). We list these genes in Table 1.

**Table 1:** **List of 39 anti-angiogenic and 89 pro-inflammatory genes**.

**Categories**	**Gene ontology**	**List of genes**
Anti-angiogenic	Negative regulation of angiogenesis (GO: 0016525)	AMOT, ANGPT2, APOH, BAI1, CCL2, CCR2, COL4A2, COL4A3, CXCL10, FASLG, FOXO4, GHRL, GTF2I, HDAC5, HHEX, HOXA5, HRG, KLF4, KLK3, KRIT1, LECT1, LIF, MAP2K5, NF1, NPPB, NPR1, PDE3B, PF4, PML, PTPRM, ROCK1, ROCK2, SERPINE1, SERPINF1, STAB1, THBS1, THBS2, THBS4, TIE1
Pro-inflammatory	Positive regulation of inflammatory response (GO:0050729)	ACE, ADAM8, ADORA2B, ADORA3, AGER, AGT, AGTR1, ALOX5AP, AOC3, C3, CCL24, CCL3, CCL3L3, CCL5, CCR2, CCR5, CCR7, CD28, CD47, CLOCK, CNR1, CTSS, CX3CL1, EDNRA, EGFR, FABP4, FCER1A, FCER1G, FCGR1A, FCGR2A, FFAR3, GPRC5B, HSPD1, HYAL2, IDO1, IL12B, IL15, IL18, IL1B, IL1RL1, IL2, IL21, IL23A, IL33, IL6, IL6ST, ITGA2, JAK2, LBP, LTA, MAPK13, MIF, NLRP12, NPY5R, OSM, OSMR, PDE2A, PDE5A, PIK3CG, PLA2G2A, PLA2G4A, PLA2G7, PRKCA, PTGER3, PTGER4, PTGS2, RPS19, S100A12, S100A8, S100A9, SERPINE1, STAT5A, STAT5B, TAC1, TGM2, TLR2, TLR3, TLR4, TLR7, TLR9, TLR10, TNF, TNFRSF11A, TNFRSF1A, TNFSF11, TNFSF4, TNIP1, WNT5A, ZP3

## Results

### Drug-targets relations in angiome, immunome and arteriome of PADPIN

We collected 11,043 binary relations between the drug and drug targets from DrugBank 3.0 (Knox et al., 2011) and 3138 binary relations between the drug and associated genes of that drug, which may not be the direct targets, from PharmGKB (Whirl-Carrillo et al., 2012). By matching the genes in angiome, immunome, and arteriome with the drug targets listed in the drug-gene binary relations from DrugBank and PharmGKB, we build the complete tables of genes and repositioning drugs (Tables S1–S3). Table S1 shows 409 and 174 drug targets listed in angiome for the drugs from DrugBank and PharmGKB, respectively. We select the genes with at least one drug targeting that gene in angiome, and skip the genes without any drug-gene relations. There might be multiple drugs targeting the same drug target; we list the multiple drugs in the same row of the table. Table S2 shows 865 and 382 drug targets in immunome for the drugs from DrugBank and PharmGKB, respectively. Table S3 shows 82 and 46 drug targets in arteriome for the drugs from DrugBank and PharmGKB, respectively.

We rank the genes in angiome, immunome, and arteriome by the degree of nodes, i.e., number of links of the nodes in the network, in Tables S1–S3, respectively. Tables S1–S3 provide the complete list of drugs and drug targets which are annotated in angiogenesis, immune response/inflammation, and arteriogenesis. Tables S1–S3 provide the complete list of drugs in DrugBank and PharmGKB, including approved, experimental, nutraceutical, illicit, and withdrawn drugs. Considering the drug safety and efficacy issues, we mostly consider the FDA-approved drugs in the predictions of repositioning drugs (Table S4).

### Inhibition of anti-angiogenic pro-inflammatory genes

We postulate two strategies to the PAD treatment: pro-angiogenic and anti-inflammatory. Starting from the 39 genes annotated in negative regulation of angiogenesis (see Materials and Methods), we match the genes with drug targets and drugs listed in Table S1, and only list the FDA-approved drugs from DrugBank in Table 2. The five genes are CCL2, NPPB, NPR1, PF4, and SERPINE1. These drugs include mimosine targeting CCL2, carvedilol targeting NPPB, nitroprusside targeting NPR1, urokinase, reteplase, and drotrecogin alfa targeting SERPINE1. The beta-blockers (e.g., carvedilol in our prediction) in general have not been shown to affect PAD symptoms, but they do not make PAD symptoms worse (Paravastu et al., 2013). Infusion of recombinant-based plasminogen activator (e.g., reteplase) and urokinase can clear blood clots and restore blood flow in occluded blood vessels of patients with diseases such as myocardial infarction and PAD (Lippi et al., 2013). The mechanism of mimosine targeting CCL2 in PAD is not clear.

**Table 2:** **Predictions of pro-angiogenic FDA-approved drugs that target anti-angiogenic genes**.

**Gene symbol**	**Gene name**	**DrugBank**	**Physiological relevance in PAD or CAD**
CCL2	Chemokine (C-C motif) ligand 2	Mimosine, danazol	Potential indicator of atherosclerosis in PAD (Rull et al., 2011)
NPPB	Natriuretic peptide B	Carvedilol	Three SNPs at NPPB locus associated with lower risk of PAD (Hu et al., 2013)
NPR1	Natriuretic peptide receptor 1	Nitroprusside, nitroglycerin, isosorbide dinitrate, amyl nitrite, erythrityl tetranitrate, nesiritide	
PF4	Platelet factor 4	Drotrecogin alfa	PF4 level increasing in patients with coronary artery ectasia (Yasar et al., 2007)
SERPINE1	Serpin peptidase inhibitor, clade E (nexin, plasminogen activator inhibitor type 1), member 1	Alteplase, urokinase, reteplase, anistreplase, tenecteplase, drotrecogin alfa	Plasminogen activator inhibitor-1 (PAI-1) increasing in patients with CLI (critical limb ischemia), leading to prothrombotic (Björck et al., 2013)

These anti-angiogenic proteins may not have direct physiological relevance in PAD. We use PubMed by searching the keywords “(Gene symbol) AND (PAD OR coronary arterial disease)” to find the relevant literature in the recent 10 years (2005–2015) for the five genes in PAD. These references support the potential biomarkers or drug targets of the five anti-angiogenic proteins in PAD, such as CCL2 (Rull et al., 2011), NPPB (Hu et al., 2013), and SERPINE1 (Yasar et al., 2007). However, these references do not link the anti-angiogenic properties of these genes to PAD. Thus, the concept of inhibition of anti-angiogenic proteins in PAD is novel and should be further explored.

### Inhibition of pro-inflammatory genes

We match the 89 pro-inflammatory genes with drug targets and drugs listed in Table S2, and only list the FDA-approved drugs from DrugBank in Table 3 (see the list of pro-inflammatory genes in Methods). The corresponding FDA-approved drugs include maraviroc (an antiretroviral drug, a CCR5 inhibitor), bosentan (a dual endothelin receptor antagonist that affects both endothelin A and B receptors, used in the treatment of pulmonary artery hypertension), sitaxentan (endothelin A receptor antagonist, used in the treatment of pulmonary artery hypertension), cetuximab (EGFR antagonist, used in several types of cancer) and imiquimod (an immune response modulator, used for skin diseases including skin cancer).

**Table 3:** **Predictions of anti-inflammatory FDA-approved drugs that target pro-inflammatory genes**.

**Gene symbol**	**Description**	**DrugBank**	**Physiological relevance in PAD or CAD**
ACE	Angiotensin I converting enzyme	Ramipril, fosinopril, trandolapril, benazepril, enalapril, candoxatril, moexipril, lisinopril, perindopril, quinapril, rescinnamine, captopril, cilazapril, spirapril	ACE inhibitor helping the walking ability in patients with CLI, but not improving ABI (ankle-pressure index) (Hunter et al., 2013; Shahin et al., 2013)
ADORA2B	Adenosine A2b receptor	Theophylline, adenosine, enprofylline, defibrotide	
AGTR1	Angiotensin II receptor, type 1	Valsartan, olmesartan, losartan, candesartan, eprosartan, telmisartan, irbesartan, forasartan, saprisartan, tasosartan	Correlation between the increased AGTR1 and cardiovascular risk factors (Baños et al., 2011)
AOC3	Amine oxidase, copper containing 3	Phenelzine, hydralazine	
C3	Complement component 3	Intravenous immunoglobulin	C3 level in serum associated with ABI and atherosclerosis in PAD patients (Fehervari et al., 2014)
CCR5	Chemokine (C-C motif) receptor 5	Maraviroc	A treatment target in pulmonary arterial hypertension (Amsellem et al., 2014)
CNR1	Cannabinoid receptor 1 (brain)	Dronabinol, nabilone, rimonabant, dronabinol	
EDNRA	Endothelin receptor type A	Bosentan, sitaxentan	
EGFR	Epidermal growth factor receptor	Cetuximab, trastuzumab, lidocaine, gefitinib, erlotinib, lapatinib, panitumumab	
FCER1A	Fc fragment of IgE, high affinity I, receptor for; alpha polypeptide	Omalizumab, benzylpenicilloyl polylysine	
FCER1G	Fc receptor, IgE, high affinity I, gamma polypeptide	Benzylpenicilloyl polylysine	
FCGR1A	Fc fragment of IgG, high affinity Ia, receptor (CD64)	Cetuximab, etanercept, intravenous immunoglobulin, adalimumab, abciximab, gemtuzumab ozogamicin, trastuzumab, rituximab, basiliximab, muromonab, ibritumomab, tositumomab, alemtuzumab, alefacept, efalizumab, natalizumab, palivizumab, daclizumab, bevacizumab, porfimer	
FCGR2A	Fc fragment of IgG, low affinity IIa, receptor (CD32)	Cetuximab, etanercept, intravenous immunoglobulin, adalimumab, abciximab, gemtuzumab ozogamicin, trastuzumab, rituximab, basiliximab, muromonab, ibritumomab, tositumomab, alemtuzumab, alefacept, efalizumab, natalizumab, palivizumab, daclizumab, bevacizumab	
IL1B	Interleukin 1, beta	Minocycline, gallium nitrate, canakinumab	
IL6	Interleukin 6	Ginseng	Phase III clinical trial targeting IL-6 by tocilizumab in cardiovascular disease (Ridker and Lüscher, 2014)
LTA	Lymphotoxin alpha	Etanercept	Implicated in predisposition for heart attack by genome-wide association studies (GWAS) (Topol et al., 2006)
PDE5A	phosphodiesterase 5A, cGMP-specific	Sildenafil, theophylline, pentoxifylline, tadalafil, vardenafil, dipyridamole, udenafil	PDE5 inhibition promotes ischemia-induced angiogenesis (Sahara et al., 2010)
PLA2G2A	Phospholipase A2, group IIA (platelets, synovial fluid)	Indomethacin, diclofenac, ginkgo biloba, suramin, ginkgo biloba	
PLA2G4A	Phospholipase A2, group IVA (cytosolic, calcium-dependent)	Fluticasone propionate, quinacrine	
PRKCA	Protein kinase C, alpha	Phosphatidylserine, vitamin E	The key protein in regulation of platelet function and thrombosis in arteries (Konopatskaya and Poole, 2010)
PTGER3	Prostaglandin E receptor 3 (subtype EP3)	Bimatoprost, dinoprostone, misoprostol	
PTGS2	Prostaglandin-endoperoxide synthase 2 (prostaglandin G/H synthase and cyclooxygenase)	Gamma-homolinolenic acid, icosapent, aminosalicylic acid, mesalazine, acetaminophen, indomethacin, nabumetone, ketorolac, tenoxicam, lenalidomide, celecoxib, tolmetin, piroxicam, fenoprofen, diclofenac, sulindac, flurbiprofen, etodolac, mefenamic acid, naproxen, sulfasalazine, phenylbutazone, meloxicam, carprofen, diflunisal, suprofen, salicyclic acid, meclofenamic acid, acetylsalicylic acid, bromfenac, oxaprozin, ketoprofen, balsalazide, thalidomide, ibuprofen, lumiracoxib, magnesium salicylate, salicylate-sodium, salsalate, trisalicylate-choline, ginseng, antrafenine, antipyrine, tiaprofenic acid, etoricoxib, niflumic acid, lornoxicam, nepafenac, gamma-homolinolenic acid, icosapent, ginseng, thalidomide	PTGS2 (COX2) inhibition improves inflammation and endothelial dysfunction in PAD patients with intermittent claudication (IC) (Flórez et al., 2009)
S100A12	S100 calcium binding protein A12	Olopatadine, amlexanox	The potential biomarker for chronic arterial disease (Saito et al., 2012) and associated with PAD (Shiotsu et al., 2011)
SERPINE1	Serpin peptidase inhibitor, clade E (nexin, plasminogen activator inhibitor type 1), member 1	Alteplase, urokinase, reteplase, anistreplase, tenecteplase, drotrecogin alfa	Level of plasminogen activator inhibitor-1 (PAI-1) increased in patients with CLI (critical limb ischemia) (Björck et al., 2013)
STAT5B	Signal transducer and activator of transcription 5B	Dasatinib	
TLR2	Toll-like receptor 2	Ospa lipoprotein	TLR2 and TLR4 expression increase during atherosclerosis, but only TLR4 gene expression associated with PAD (Varela et al., 2015)
TLR7	Toll-like receptor 7	Imiquimod, hydroxychloroquine	
TLR9	Toll-like receptor 9	Chloroquine, hydroxychloroquine	
TNF	Tumor necrosis factor	Etanercept, adalimumab, infliximab, chloroquine, thalidomide, glucosamine, clenbuterol, pranlukast, amrinone, thalidomide	Circulating TNF higher in PAD patients than control (Gardner et al., 2014); Circulating cytokines induce endothelial dysfunction in PAD patients (Botti et al., 2012); Negative correlation between TNF concentration and pain free walking distance (Wozniak et al., 2012)
TNFSF11	Tumor necrosis factor (ligand) superfamily, member 11	Lenalidomide	

To find the physiological relevance of these pro-inflammatory genes in PAD, we continue to use PubMed to find the relevant references. References in Table 3 support our hypothesis that anti-inflammatory drugs have high potential for repositioning for PAD. Some drugs cannot improve ABI (ankle-pressure index) of PAD patients but can improve the walking ability in patients with critical limb ischemia (CLI), such as ACE inhibitors (Hunter et al., 2013; Shahin et al., 2013). Some genes are indicated as related with PAD, such as C3 (complement component 3) (Fehervari et al., 2014), PTGS2 (prostaglandin-endoperoxide synthase 2) (Flórez et al., 2009), SERPINE1 (Björck et al., 2013), S100A12 (Shiotsu et al., 2011), and TNF (Botti et al., 2012; Wozniak et al., 2012; Gardner et al., 2014). Some genes are potential biomarkers or associated with other cardiovascular diseases, such as AGTR1 (angiotensin II receptor, type 1) in coronary occlusive disease (Baños et al., 2011), CCR5 in pulmonary arterial hypertension (Amsellem et al., 2014), LTA (lymphotoxin alpha) in CAD (Topol et al., 2006), and PRKCA (protein kinase C, alpha) in atherosclerosis (Konopatskaya and Poole, 2010). Many of the anti-inflammatory genes in Table 3 are not directly associated with PAD or CAD based on PubMed search, such as ADORA2B (adenosine A2b receptor), EDNRA (endothelin receptor type A), FCER1G (Fc receptor, IgE, high affinity I, gamma polypeptide), STAT5B (signal transducer and activator of transcription 5B), and TLR9 (toll-like receptor 9). In general, the physiological evidence of these anti-inflammatory genes listed in Table 3 strongly supports our hypothesis that inhibition of pro-inflammatory genes is a viable drug repositioning strategy in PAD.

### Visualization of drug-target network

**Figure 1:**
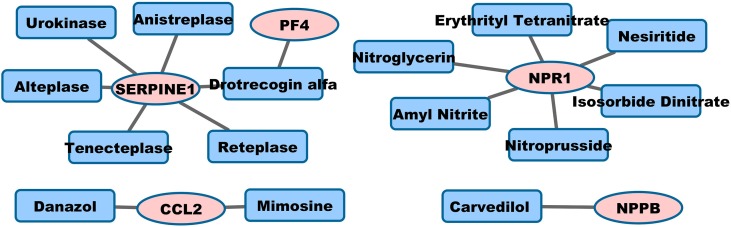
**Pro-angiogenic drug-target interaction networks**.

**Figure 2:**
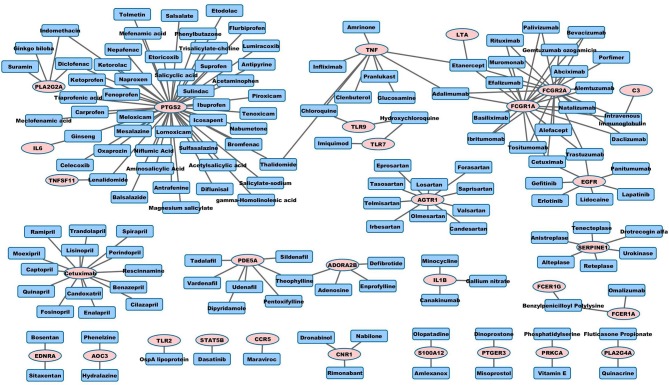
**Anti-inflammatory drug-target interaction networks**.

Graph representation is used to visualize pro-angiogenic and anti-inflammatory repositioning drugs for PAD in Figures 1, 2, respectively. We plot the drug-target networks of the anti-angiogenic and pro-inflammatory proteins for the drugs in Tables 2, 3, respectively. We represent the drug target by pink circle and the drug by blue square. Figure 1 shows several compounds targeting the proteins which are annotated as negative regulation of angiogenesis. Figure 2 shows the drug-target networks of the anti-inflammatory drugs and targets from Table 3. The number of inflammation targets and drugs in Figure 2 is much larger than anti-angiogenic targets and drugs in Figure 1. This gives the insight for the development of clinical trials of anti-inflammatory drugs in PAD in the future. We will discuss the potential clinical trials in Discussion.

## Discussion

The clinical trials aimed at stimulating VEGF in PAD and CAD have been unsuccessful (Annex, 2013). The exercise therapy has been demonstrated as the beneficial treatment for PAD, including walking tolerance, modified inflammatory markers, and adaptation of the limb (e.g., angiogenesis and arteriogenesis) (Haas et al., 2017). Clinical trials with agents targeting angiogenesis and inflammation, other than stimulation of VEGF, should be considered in the future. Below we provide insights for the potential repositioning drugs in PAD identified in this study, including the mechanism of action of these drugs, case studies for several selected drugs in clinical trials, and future experimental validations.

### Mechanism of action of repositioning drugs for PAD

Tables 1, 2 provide the anti-angiogenic and pro-inflammatory genes/proteins, drugs targeting these molecules, and physiological evidence for the involvement of these molecules in PAD. However, even though these drug-targets have been identified by our bioinformatics approaches, the mechanism of action of these drugs in PAD and the feasibility of the clinical trials need to be elucidated. Specifically, the effect of some of these drugs to promote angiogenesis in PAD by targeting anti-angiogenic proteins is unknown. Therefore, we search PubMed for the drugs listed in Table 2 using the keywords “(drug name) AND angiogenesis” to understand the mechanism and original use of these putative pro-angiogenic drugs. We list the drugs with at least one supporting reference found in PubMed in Table 4. These drugs include beta-1 adrenergic receptors blocker (carvedilol, targeting NPPB), vasodilator (isosorbide dinitrate, targeting NPR1), and plasminogen activator (alteplase, targeting SERPINE1).

**Table 4:** **Mechanism of Action and original use of the drugs for repositioning as pro-angiogenic in PAD**.

**Drug name**	**Target in angiogenesis**	**Degree of target in angiome**	**Mechanism of action**	**Original use**	**PubMed search**
Alteplase	SERPINE1	14	Plasminogen activator	Acute ischemic stroke	Lapchak and Araujo, 2007; Hacke et al., 2008
Danazol	CCL2	12	Synthetic steroid with antigonadotropic and anti-estrogenic activities	Endometriosis	Thomas et al., 2007; Szubert et al., 2014
Nitroprusside	NPR1	4	A source of nitric oxide, a potent peripheral vasodilator	Hypertensive emergency	Ziche et al., 1994; Pyriochou et al., 2007
Isosorbide dinitrate	NPR1	4	Vasodilator	Angina pectoris	Goertz et al., 2011
Nesiritide	NPR1	4	Recombinant form of brain natriuretic peptide	Heart failure	Shmilovich et al., 2009
Carvedilol	NPPB	3	Beta-1 and beta-2 adrenergic receptors blocker	Congestive heart failure	Le et al., 2013; Stati et al., 2014

We further search PubMed for the anti-inflammatory drugs in Table 3 using the keywords “(drug name) AND inflammation” to elucidate the mechanism and original use of these anti-inflammatory drugs (Table 5). These drugs include antiplatelet (abciximab targeting FCGR1A, acetylsalicylic acid targeting PTGS2), monoclonal antibody (adalimumab targeintg TNF-alpha), immune suppressant (alefacept targeting FCGR1A and FCGR2A), ACE inhibitor (benazepril, captopril and enalapril), non-steroidal anti-inflammatory drug (NSAID, e.g., bromfenac, celecoxib, diclofenac, ketorolac, nepafenac, sulindac), and PDE5 inhibitor (tadalafil, vardenafil).

**Table 5:** **Mechanism of Action and original use of the drugs for repositioning as anti-inflammatory in PAD**.

**Drug name**	**Target in inflammation**	**Degree of target in immunome**	**Mechanism of action**	**Original use**	**PubMed search**
Lapatinib	EGFR	137	Tyrosine kinase inhibitor	Breast cancer	Hall et al., 2009
Lidocaine	EGFR	137	Stopping nerves from sending pain signal	Local anesthetic and class-1b antiarrhythmic drug	Caracas et al., 2009
Maraviroc	CCR5	46	CCR5 receptor antagonist class	Human immunodeficiency virus (HIV) infection	Francisci et al., 2014
Dasatinib	STAT5B	33	Bcr-Abl tyrosine kinase inhibitor	Leukemia	Futosi et al., 2012
Chloroquine	TNF, TLR9	22, 22	4-aminoquinoline drug	Malaria, rheumatoid arthritis	Yang et al., 2013
Clenbuterol	TNF	22	Angiotensin-converting enzyme (ACE) inhibitor	Hypertension and heart failure	Cudmore et al., 2013
Glucosamine	TNF	22	Endogenous amino-monosaccharide synthesized from glucose	Promoting joint and cartilage health	Azuma et al., 2015; Chou et al., 2015
Infliximab	TNF	22	Chimeric monoclonal antibody against TNF alpha	Rheumatoid arthritis, psoriatic arthritis, ankylosing spondylitis	Hirono et al., 2009
Alteplase	SERPINE1	18	Plasminogen activator	Acute ischemic stroke	Lapchak and Araujo, 2007; Hacke et al., 2008
Intravenous immunoglobulin	C3, FCGR1A, FCGR2A	30, 13, 11	IgG antibodies	Immune deficiencies, autoimmune diseases	Nimmerjahn and Ravetch, 2008
Drotrecogin alfa	SERPINE1	18	Recombinant form of human activated protein C	Decrease inflammation and the formation of blood clots in blood vessels	Rice and Bernard, 2004
Candesartan	AGTR1	16	Angiotensin II receptor antagonist	High blood pressure and heart failure	Yu et al., 2007
Eprosartan	AGTR1	16	Angiotensin II receptor antagonist	Treats high blood pressure	Rahman et al., 2002
Irbesartan	AGTR1	16	Angiotensin receptor blocker (ARB)	High blood pressure	Taguchi et al., 2013
Losartan	AGTR1	16	Angiotensin receptor blocker (ARB)	High blood pressure	Merino et al., 2012
Olmesartan	AGTR1	16	Angiotensin receptor blocker	High blood pressure	Nagib et al., 2013
Telmisartan	AGTR1	16	Angiotensin receptor blocker (ARB)	High blood pressure	Al-Hejjaj et al., 2011
Valsartan	AGTR1	16	Angiotensin receptor blocker (ARB)	High blood pressure and heart failure	Wang et al., 2014
Adalimumab	FCGR1A, FCGR2A, TNF	13, 11, 22	Monoclonal antibody against TNF-alpha	Arthritis, ankylosing spondylitis	Jiang et al., 2013
Lenalidomide	TNFSF11	15	Immunomodulatory and antiangiogenic agent	Anemia and multiple myeloma	Rozovski et al., 2013
Thalidomide	PTGS2, TNF	7, 22	Immunomodulatory drug	Certain cancers, leprosy	Keifer et al., 2001
Etanercept	FCGR1A, FCGR2A, LTA, TNF	13, 11, 7, 22	Tumor necrosis factor (TNF) inhibitor	Rheumatoid arthritis, psoriatic arthritis, ankylosing spondylitis, and plaque psoriasis	Cao et al., 2015
Abciximab	FCGR1A, FCGR2A	13, 11	Inhibits platelet aggregation by preventing the binding of fibrinogen	Patients undergoing percutaneous coronary intervention (PCI)	Hong et al., 2007
Alefacept	FCGR1A, FCGR2A	13, 11	Immune suppressant	Control of inflammation in moderate to severe psoriasis	Kraan et al., 2002; Chamian et al., 2005
Alemtuzumab	FCGR1A, FCGR2A	13, 11	Binds to CD52, a protein present on the surface of mature lymphocytes	Chronic lymphocytic leukemia, multiple sclerosis	Heilman et al., 2013
Daclizumab	FCGR1A, FCGR2A	13, 11	Monoclonal antibody binding to CD25, alpha subunit of the IL-2 receptor of T cells	Prevents rejection in organ transplantation, multiple sclerosis	Papaliodis et al., 2003
Efalizumab	FCGR1A, FCGR2A	13, 11	Immunosuppressant by inhibiting lymphocyte activation	Psoriasis	Pan et al., 2014
Rituximab	FCGR1A, FCGR2A	13, 11	Monoclonal antibody against the protein CD20	Rheumatoid arthritis	Baslund et al., 2012
Canakinumab	IL1B	10	Monoclonal antibody targeted at interleukin-1 beta	Rheumatoid arthritis, coronary artery disease	Ridker et al., 2011
Gallium nitrate	IL1B	10	Gallium salt of nitric acid	Symptomatic hypercalcemia	Eby, 2005
Minocycline	IL1B	10	Bacteriostatic antibiotic	Treats infections	Leite et al., 2011
Aminosalicylic acid	PTGS2	7	Inhibits folic acid synthesis and synthesis of the cell wall component	Tuberculosis	Williams et al., 2011
Balsalazide	PTGS2	7	Converted in the body to mesalamine and reducing bowel inflammation	Ulcerative colitis	Pardi et al., 2002
Acetaminophen	PTGS2	7	Analgesics (pain relievers)	Treats minor aches and pain and reduces fever	Jeon et al., 2014
Acetylsalicylic acid (aspirin)	PTGS2	7	Antiplatelet effect by inhibiting thromboxane	Prevention of arterial and venous thrombosis	Herová et al., 2014
Bromfenac	PTGS2	7	Non-steroidal anti-inflammatory drug (NSAID)	Pain and swelling of the eye after cataract surgery	Rajpal et al., 2014
Etodolac	PTGS2	7	Non-steroidal anti-inflammatory drug (NSAID)	Treats pain caused by arthritis	Costa et al., 2008
Etoricoxib	PTGS2	7	COX-2 selective inhibitor	Rheumatoid arthritis, psoriatic arthritis, osteoarthritis	Moraes et al., 2007
Gamma-homolinolenic acid	PTGS2	7	Omega-6 fatty acid	Dietary supplement for a variety of human health problems	Kapoor and Huang, 2006
Carprofen	PTGS2	7	Reduces inflammation by inhibition of COX-2	Pain and inflammation from arthritis	Fox and Johnston, 1997
Celecoxib	PTGS2	7	COX-2 selective non-steroidal anti-inflammatory drug (NSAID)	Treats pain caused by arthritis	Chen et al., 2008
Ibuprofen	PTGS2	7	Non-steroidal anti-inflammatory drug (NSAID)	Pain and fever	Chmiel et al., 2015
Ketoprofen	PTGS2	7	Non-steroidal anti-inflammatory drugs (NSAIDs)	Relief of pain and inflammation such as in rheumatic disease	Choi et al., 2013
Ketorolac	PTGS2	7	Non-steroidal anti-inflammatory drugs (NSAIDs)	Pain and inflammation caused by arthritis	Donnenfeld et al., 2011
Lornoxicam	PTGS2	7	Non-steroidal anti-inflammatory drug	Pain, especially resulting from inflammatory diseases	Buritova and Besson, 1997, 1998
Mefenamic acid	PTGS2	7	Non-steroidal anti-inflammatory drug (NSAID)	Pain	Cunha et al., 2014
Meloxicam	PTGS2	7	Non-steroidal anti-inflammatory drug (NSAID)	Pain	
Mesalazine	PTGS2	7	5-amino-2-hydroxybenzoic acid	Inflammatory bowel disease, such as ulcerative colitis	
Nabumetone	PTGS2	7	Non-steroidal anti-inflammatory drug (NSAID)	Relief of pain and inflammation in arthritis	
Naproxen	PTGS2	7	Non-steroidal anti-inflammatory drugs (NSAIDs)	Relief of pain, fever, swelling, and stiffness	
Nepafenac	PTGS2	7	Non-steroidal anti-inflammatory drugs (NSAIDs)	Eye pain and swelling after cataract surgery	Nardi et al., 2007
Niflumic acid	PTGS2	7	Inhibitor of cyclooxygenase-2	Joint and muscular pain	Bilecen et al., 2003
Piroxicam	PTGS2	7	Non-steroidal anti-inflammatory drugs (NSAIDs)	Pain, including arthritis pain	
Salsalate	PTGS2	7	Non-steroidal anti-inflammatory drugs (NSAIDs)	Rheumatoid arthritis	Goldfine et al., 2008
Sulindac	PTGS2	7	Non-steroidal anti-inflammatory drug (NSAID)	Treats pain caused by arthritis, gout, or sore tendons	Mladenova et al., 2013
Tenoxicam	PTGS2	7	Non-steroidal anti-inflammatory drug (NSAID)	Relieve inflammation and pain associated with rheumatoid arthritis	
Indomethacin	PTGS2, PLA2G2A	7, 6	Non-steroidal anti-inflammatory drugs (NSAIDs)	Reduce fever, pain, stiffness, and swelling	Glaser et al., 1993
Suramin	PLA2G2A	6	Antimicrobial drug	Protozoa, Helminthiasis	Shiono et al., 2002; Liu et al., 2012
Bosentan	EDNRA	4	Dual endothelin receptor antagonist	Pulmonary artery hypertension	Shetty and Derk, 2011
Omalizumab	FCER1A	3	Reduce sensitivity to inhaled or ingested allergens	Asthma	Holgate et al., 2005, 2009
Enazepril	ACE	2	Angiotensin-converting enzyme (ACE) inhibitor	High blood pressure	Yan et al., 2013
Captopril	ACE	2	Angiotensin-converting enzyme (ACE) inhibitor	High blood pressure and heart failure	El Desoky, 2011
Dinoprostone	PTGER3	2	Prostaglandin E2 (PGE2)	Helps dilate the opening of the uterus (cervix) in a pregnant woman	Tang et al., 2012
Dipyridamole	PDE5A	2	Inhibits the phosphodiesterase enzymes, coronary vasodilator	Inhibits thrombus formation	Weyrich et al., 2005; Massaro et al., 2013
Enalapril	ACE	2	Angiotensin converting enzyme (ACE) inhibitors	Treats high blood pressure	da Cunha et al., 2005
Misoprostol	PTGER3	2	Non-steroidal anti-inflammatory drugs (NSAIDs)	Prevents stomach ulcers	Rossetti et al., 1995
Pentoxifylline	PDE5A	2	Phosphodiesterase inhibiton	Treating intermittent claudication resulting from peripheral artery disease	Abdel-Salam et al., 2003; Fernandes et al., 2008
Perindopril	ACE	2	ACE inhibitor	Treats high blood pressure and coronary artery disease	Rowbotham et al., 1996
Quinapril	ACE	2	ACE inhibitor	High blood pressure and heart failure	Egido and Ruiz-Ortega, 2007
Bildenafil	PDE5A	2	Inhibiting cGMP-specific phosphodiesterase type 5	Erectile dysfunction, pulmonary arterial hypertension	de Visser et al., 2009
Tadalafil	PDE5A	2	PDE5 inhibitor	Erectile dysfunction, benign prostatic hyperplasia, pulmonary arterial hypertension	Varma et al., 2012
Theophylline	PDE5A, ADORA2B	2, 2	Methylxanthine drug	Asthma and respiratory disease	Cosio et al., 2009
Vardenafil	PDE5A	2	PDE5 inhibitor	Erectile dysfunction	Lubamba et al., 2012
Hydralazine	AOC3	1	Vasodilator	Treats high blood pressure and heart failure	Bai et al., 2012

### Case studies of potential drug targets and drug repositioning in PAD

We choose three candidate drugs for repositioning in PAD as case studies of our predictions. We selected several drugs that are anti-inflammatory or pro-angiogenic and had no effects on each other. These drugs include bosentan, carvedilol, and maraviroc. We compared the drug targets with the up-regulated genes in the microarray dataset of PAD, including the mouse data of Hazarika et al. (2013), and human microarray studies of Masud et al. (2012), Fu et al. (2008), and Croner et al. (2012).

#### Case I: Bosentan targeting EDNRA

The endothelin receptor antagonists (bosentan and ambrisentan) have been approved for use in pulmonary arterial hypertension (PAH) and have been assigned orphan drug status. Details of hepatotoxicity of bosentan, ambrisentan, and sitaxentan are reviewed in de Haro Miralles et al. (2010). Endothelin-1 is a powerful endogenous vasoconstrictor (Frumkin, 2012) and thus blocking endothelin could improve perfusion to the lower extremities in patients with PAD. In a pre-clinical PAD model, Luyt et al. (2000) demonstrated that endothelin, antagonists, bosentan, and darusentan (LU13525) increased tissue blood flow measured by laser Doppler perfusion imaging. de Haro Miralles et al. (2010) examined plasma levels of endothelin and showed that endothelin levels were increased in patients with intermittent claudication compared to non-PAD controls. Just as importantly patients with the most severe form of PAD, CLI, did not demonstrate elevated levels of endothelin, which suggests that an elevation of endothelin is specific to the pathophysiology of intermittent claudication and not all forms of PAD. The original indication of zibotentan was in oncology and pulmonary artery hypertension. The reuse of an endothelin receptor antagonists in PAD patients with intermittent claudication is now in a Phase II clinical trial; the details of the clinical trial of zibotentan are provided in ClinicalTrials.gov https://clinicaltrials.gov/ct2/show/NCT01890135?term=NCT01890135&rank=1.

#### Case II: Carvedilol targeting NPPB

Carvedilol has anti-inflammatory and pro-angiogenic effects in chronic ischemic cardiomyopathy (Le et al., 2013). Carvedilol showed improvement of myocardial flow and reduction of inflammation in the canine model of multivessel cardiomyopathy. The anti-inflammatory cytokine IL-10, which inhibits inflammatory cytokines such as TNFα, IL-1, IL-6, IL-8, and IL-12, was up-regulated in the carvedilol-treated animals. In the PAD microarray data, the inflammatory cytokine IL-8 was up-regulated as found in Masud et al. (2012) and Croner et al. (2012). Though beta-blockers are commonly used in patients with PAD, currently there are no specific clinical trials for carvedilol being compared to placebo or other beta-blockers in PAD patients.

#### Case III: Maraviroc targeting CCR5

Maraviroc is an HIV drug targeting CCR5, which is involved in the inflammation pathway (Francisci et al., 2014). Therefore, maraviroc could have anti-inflammatory and anti-atherosclerosis effects, and become a potential repositioning drug in PAD. Croner et al. (2012) show the up-regulation of CCR5 in microarrays from the human femoral artery in PAD. CCR5 inhibitor maraviroc also blocks cell migration and metastasis, but not directly affects the angiogenesis pathway in triple negative breast cancer cell lines (Lee et al., 2014). Currently there are no clinical trials for maraviroc in PAD patients.

### Limitations of computational drug repositioning approaches

There are several limitations by the computational approaches to predict the repositioning drugs in PAD. First, PAD is a complex disease caused by many risk factors and classified by different stages of diseases. Our methods cannot predict the repositioning drugs based on various conditions in PAD patients. Second, the current available clinical trials based on these predicted repositioning drugs in PAD patients are very limited. The available gene expression dataset in human PAD and mouse PAD model is limited. It is difficult to validate our predictions by current clinical trials and available microarray data. Third, the pro-angiogenic and anti-inflammatory drug-target networks cannot directly link the drugs to PAD based on the current physiological evidence in PAD. The value of the computational drug repositioning might be limited for clinical trial design.

## Conclusions

Our study provides comprehensive predictions of potential pro-angiogenic and anti-inflammatory drugs and drug targets for PAD patients. Based on the protein-protein interaction network PADPIN, we collected the binary relations between FDA-approved drugs and genes annotated in PADPIN. By gathering FDA-approved drugs, these predictions form a basis for further validation and future translational research in PAD.

### Conflict of interest statement

The authors declare that the research was conducted in the absence of any commercial or financial relationships that could be construed as a potential conflict of interest.
